# Chloroquine Protects Human Corneal Epithelial Cells from Desiccation Stress Induced Inflammation without Altering the Autophagy Flux

**DOI:** 10.1155/2018/7627329

**Published:** 2018-11-01

**Authors:** Shivapriya Shivakumar, Trailokyanath Panigrahi, Rohit Shetty, Murali Subramani, Arkasubhra Ghosh, Nallathambi Jeyabalan

**Affiliations:** ^1^GROW Research Laboratory, Narayana Nethralaya Foundation, Bangalore, Karnataka, India; ^2^Department of Cornea and Refractive surgery, Narayana Nethralaya Eye Hospital, Narayana Health City, Bommasandra, Bangalore, Karnataka, India

## Abstract

Dry eye disease (DED) is a multifactorial ocular surface disorder affecting millions of individuals worldwide. Inflammation has been associated with dry eye and anti-inflammatory drugs are now being targeted as the alternate therapeutic approach for dry eye condition. In this study, we have explored the anti-inflammatory and autophagy modulating effect of chloroquine (CQ) in human corneal epithelial and human corneal fibroblasts cells exposed to desiccation stress, (an* in-vitro* model for DED). Gene and protein expression profiling of inflammatory and autophagy related molecular factors were analyzed in HCE-T and primary HCF cells exposed to desiccation stress with and without CQ treatment. HCE-T and HCF cells exposed to desiccation stress exhibited increased levels of activated p65, TNF-*α*, MCP-1, MMP-9, and IL-6. Further, treatment with CQ decreased the levels of active p65, TNF-*α*, MCP-1, and MMP-9 in cells underdesiccation stress. Increased levels of LC3B and LAMP1 markers in HCE-T cells exposed to desiccation stress suggest activation of autophagy and the addition of CQ did not alter these levels. Changes in the phosphorylation levels of MAPKinase and mTOR pathway proteins were found in HCE-T cells under desiccation stress with or without CQ treatment. Taken together, the data suggests that HCE-T cells under desiccation stress showed NF*κ*B mediated inflammation, which was rescued through the anti-inflammatory effect of CQ without altering the autophagy flux. Therefore, CQ may be used as an alternate therapeutic management for dry eye condition.

## 1. Introduction

Dry eye disease (DED) is characterized by an abnormal instability of the tear film leading to ocular discomfort, visual disturbance, inflammation, dryness, and irritation of the eye [1]. Inflammation is a primary response of one's body towards stress and foreign substances; it is a major risk factor for several chronic diseases including dry eye. Recent studies have reported that inflammation associated with dry eye was mediated by T-lymphocytes [2]. Dry eye patients were found with higher levels of proinflammatory cytokines such as IL1*α* [3] along with IL-1*β*, IL-6, IL-8, and tumor necrosis factor (TNF-*α*) in the tear film compared to normal controls [4]. Chloroquine (CQ), a widely known antimalarial drug has been used as an anti-inflammatory agent for treating rheumatoid arthritis [5] and discoid lupus erythematosus [6]. Studies have shown that CQ inhibits the release of pro-inflammatory cytokines in the human blood during chronic and bacteria-induced inflammation [7]. CQ blocks TNF-*α* and IL-6 synthesis in lipopolysaccharide (LPS) stimulated inflammation in mouse macrophages. It was also been found to inhibit LPS-induced activation of TNF-*α*  & ERK1/2 gene expression in PBMC's [8]. In addition to its anti-inflammatory activity CQ is a known modulator of autophagy. Autophagy is a cellular mechanism through which damaged intracellular molecules and organelles are cleared through the autophagic lysosomal pathway. It is activated during stress and starvation, thereby allowing cells to survive in response to various stresses [9]. Autophagy has been implicated in a number of fundamental biological processes including aging, immunity, inflammation, development, and differentiation [10]. Studies have reported higher doses of CQ with prolonged oral administration leading to retinal toxicity, with decreased visual acuity, diplopia, and bilateral vision loss [11], and other additional side effects such as ototoxicity, seizures, insomnia, and paresthesia [12]. Anti-inflammatory drugs like cyclosporine and chloroquine are widely being used as alternate therapy for dry eye condition. Clinical application of CQ showed anti-inflammatory effect through the improvement in immunoglobulin levels and erythrocyte sedimentation rate (ESR) and decrease in inflammatory cytokines in primary Sjogren's syndrome (pSS). However, the clinical efficacy was is moderate in keratoconjunctivitis sicca syndrome [13, 14].

In spite of the known anti-inflammatory and autophagy modulating activity of CQ, its modulation on ocular surface disease mechanisms has not been explored. In addition, studies have shown that inflammation and autophagy are interdependent process [15], but the underlying mechanisms still remain unclear. Therefore, to explore the therapeutic effect of CQ in the pathogenesis of dry eye condition, we subjected primary human corneal epithelial cells, HCE-T cells, and corneal fibroblast (HCF) cells to desiccation stress (experimental condition for dry eye) and analysed the anti-inflammatory and autophagy modulating activity of CQ. Several studies have shown direct association and increased expression levels of inflammatory cytokines IL-6, TNF-*α* [16], MCP-1 [17], and MMP-9 in tears of dry eye patients. Hence, we are interested in studying the effect of CQ on the above cytokines levels in* in-vitro* experimental conditions for dry eye disease.

## 2. Materials and Methods

### 2.1. Viability Assay for HCE Cells Treated with CQ

HCE-T cells were treated with different concentrations (0.00006 to 0.003%) of CQ for 48 hrs. Tryphan blue assay was used to determine the cell viability.

### 2.2. Immunofluorescence Staining

HCE-T cells were cultured on chamber slides at density of 0.1 × 10^6^ cells/well. After 24 hours the media were removed and cells were fixed with 100% ice cold methanol for 5 minutes at room temperature. Further, cells were treated with permeabilization buffer containing 1XPBS and 0.1% triton X-100. Cells were then blocked with 3% bovine serum albumin (BSA) at room temperature for 30 minutes, followed by incubation with primary cytokeratin 3 antibody (abcam, Cat no- ab77869) (1:500) overnight at 4 degree. Alexa fluor 488- conjugated anti-mouse secondary antibody (abcam, Cat no- ab150113) was used (1:2000) and kept for 1 hour incubation at room temperature. Finally the cells were mounted using fluoroshield containing DAPI (Fluoroshield™ sigma, cat no- F6057) and examined under fluorescence microscope using FL1 and FL2 channels.

### 2.3. Cell Culture and Desiccation Stress

Primary human corneal epithelial cells (HCE) of limbal origin were derived from donor corneal tissues and cultured according to the protocol [18]. Human corneal fibroblasts (HCF) cells were derived from donor corneal buttons by following previously mentioned protocol [19]. SV40 large T antigen immortalized human corneal epithelial cell line (HCE-T) and HCF cells (passage 3) were cultured at the density of 0.3 × 10^6^ cells/well in a growth medium (DMEM/F-12, Gibco, USA) containing 5% and 20% fetal bovine serum (Gibco, USA), 100 U/ml penicillin, and 100 mg/ml streptomycin sulphate (Sigma-Aldrich, St. Louis, MO) at 37°C. To induce desiccation stress, the media were completely aspirated from primary HCE, HCF, and HCE-T cells and air dried for 10 minutes at room temperature (25°C) and humidity of (40%). Further, the growth media were replenished and cells were treated with 0.03% chloroquine (CQ- UV LUBE UNIMS – FDC Ltd., India) (5 *μ*l/ml) and Restasis 0.05% (CsA - Cyclosporine ophthalmic emulsion, Allergan, Pvt. Ltd, India) (2 *μ*l/ml). After 24 hrs, HCE-T cells and primary HCE and HCF cells exposed to desiccation stress were evaluated for morphology and viability using light microscopy and trypan blue assays, respectively.

### 2.4. RNA Isolation and qPCR Analysis

Total RNA was extracted from HCE-T and HCF cells exposed to desiccation stress, treated with and without CQ/CsA using Trizol reagent (Invitrogen, USA). RNA was quantified using a nanodrop spectrophotometer (NanoDrop 1000, Thermo Scientific, DE, USA). 1000 ng of the total RNA was converted to cDNA using Biorad iScript™ cDNA synthesis kit. Quantitative real-time PCR was performed using 4 *μ*l of 10-fold diluted cDNA in a final volume of 10 *μ*l using the SYBR Green master mix (Bio-Rad, Philadelphia, PA, USA). Gene expression profile was studied for inflammatory and autophagy related genes- IL-6, TNF-*α*, MCP-1, MMP-9, LC3A, LC3B, LAMP1, and ATG7. The primer sequences used are mentioned in Table 1. All data was analyzed by ΔΔCt method and the mRNA of beta-*actin* was used as the internal standard.

### 2.5. Protein Extraction and Western Blot Analysis

Whole cell protein lysate and nuclear-cytoplasmic fractions were extracted from HCE-T and HCF cells exposed to desiccation stress, treated with and without CQ/CsA using RIPA lysis buffer and nuclear/cytoplasmic extraction kit respectively (Cat no-786-489 and 786-182, G-Biosciences, USA). Protease and phosphatase inhibitors [(phosphostop cat no- 0490687001, cOmplete EDTA free protease inhibitor cocktail (Cat no-04693159001, Roche life science, USA)] were added to the extraction buffers. The cells were snap frozen and thawed twice followed by vortexing for 30 sec which was repeated five times. The lysate was centrifuged at 13000 rpm for 20 minutes at 4°C. Protein concentration was measured using Bradford reagent (Cat no-5000006, Biorad, USA). 20 *μ*g of protein was loaded & run on 10% SDS PAGE, blotted onto PVDF membrane and blocked with 5% milk powder in TBST for 1hr at room temperature. All primary antibodies were incubated overnight in dark at 4 degree: p65 [1:1000, Cell Signaling-C20], P-p65 (Ser536) [1:1000, Cell Signaling-(93H1)], I*κ*B*α* [1:1000, Cell Signaling (L35A5)], LAMP1 [1:1000, Cell Signaling (D2D11) XP], LC3A/B [1:1000, Cell Signaling, (#4108)], SQSTM1/p62 [1:1000,Cell Signaling (#5114)], p38 [1:1000, Cell signalling (#9202)], P-p38 (Thr180/Thy182) [1:1000, Cell Signaling (3D7)], p70S6Kinase [1:1000, Cell Signaling (9202)], P-p70S6Kinase (Thy389) [1:1000, Cell Signaling (9205)], ERK1/2 [1:1000, Cell signalling (137F5)], P-ERK1/2 (Thr180/Thy204) [1:1000, Cell signalling (D13.14.4E)], Akt [1:1000, Cell Signaling (4691)], P-Akt (Ser473) [1:1000, Cell Signaling (9271)], Beclin-1 [1:1000, Cell signalling (D40C5)] *β*- actin [1:3000, Santa Cruz, C-4], and GAPDH [1:2000, Abgenex, Clone:ABM22C5]. The secondary antibodies (anti-rabbit, anti-mouse) were conjugated with horseradish peroxidase and a chemiluminescence substrate (Biorad, Philadelphia, PA, USA) to visualize the band (Image Quant LAS 500, GE Healthcare, Life Sciences, USA).

### 2.6. Transfection

HCE-T cells were cultured in chamber slide at a density of 10000 cells/well and transfected with GFP-RelA plasmid (Addgene, Plasmid #23255) using Xfect™ transfection reagent (Cat no- 631317, Clontech, Takara, USA.) as per manufacturer's protocol. After 24 hrs, cells were exposed to desiccation stress followed by treatment with and without CQ/CsA. TNF-*α* ((10 ng/ml); Cat no-654205, Calbiochem, Merk, Germany) was used as a positive control to observe the GFP-RelA nuclear translocation. The localisation of GFP-RelA in HCE-T cells exposed to desiccation stress withand without CQ/CsA treatment was analysed under fluorescence microscope (EVOS -FL- Auto Cell Imaging System, Thermo fisher Scientific, USA).

### 2.7. Fluorescence Staining

HCE-T cells were cultured on 0.3% gelatin coated cover slips at a density of 0.3 × 10^6^ cells/well. Then cells were exposed to desiccation stress, treated with and without CQ/CsA. Cells were then incubated with CYTO-ID and Lysotracker dye (LTR) to quantify autophagosome and lysosomes followed by the method as described previously [20]. The lysosomal pH of HCE-T cells exposed to desiccation stress was assessed using acridine orange dye. Cells were fixed with 4% paraformaldehyde and examined under fluorescence microscope.

### 2.8. Statistical Analysis

Experiments were performed three independent times and the data are represented as a mean ± SD (n = 3). One way ANOVA followed by Dunnett's multiple analysis was performed using Graphpad prism software version 6. The level of significance is represented as (^*∗*^P < 0.05, ^*∗∗*^P < 0.01, and ^*∗∗∗*^P < 0.001).

## 3. Results

### 3.1. Cytotoxic Activity of CQ on HCE Cells

HCE-T cells treated with different concentrations of CQ (0.00006 to 0.003%) for 48 hrs were analysed for cell viability using trypan blue dye. The cells treated with 0.00006% of CQ showed 3% cell death and 80-84% was observed at 0.003% (Supplementary figure 1).

### 3.2. Cytokeratin 3 Staining in HCE Cells

Immunostaining of cytokeratin 3 in HCE-T cells showed presence of green fluorescence in the cytoplasm region, with the typical sign of filamentous staining emerging from the nucleus towards the cell membrane (Supplementary figure 2).

### 3.3. HCE and HCF Cells under Desiccation Stress

**Figure 1:**
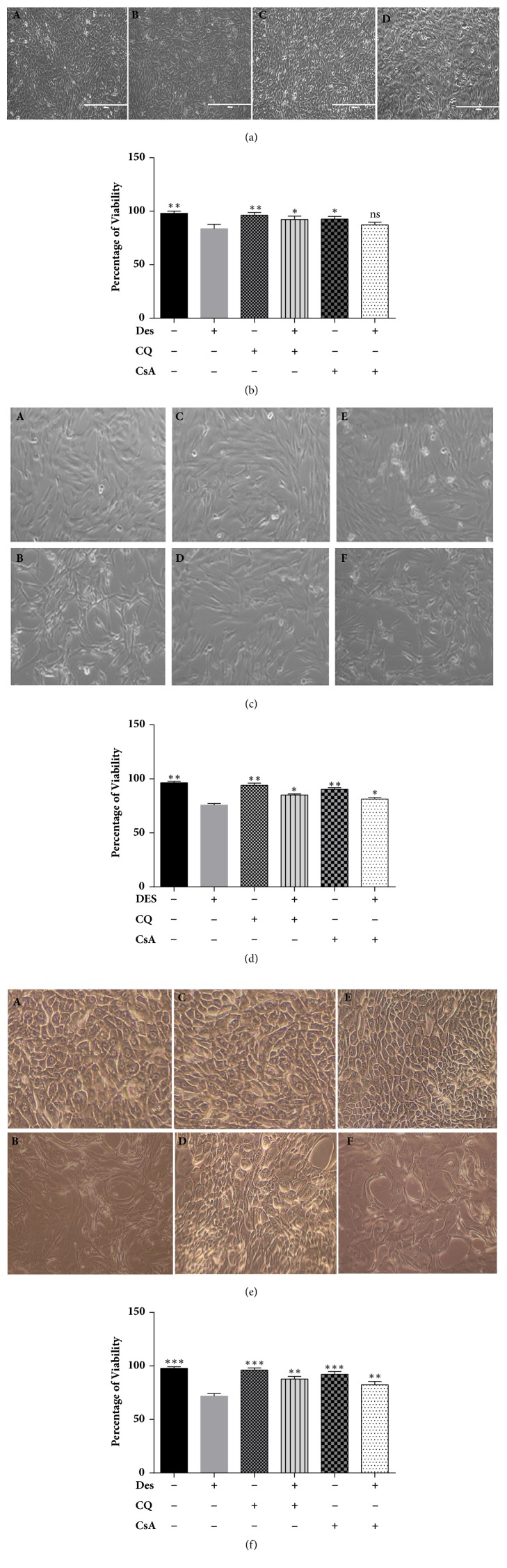
**Morphological and viability analysis of HCE-T, Primary HCF, and HCE cells exposed to desiccation stress**. (a) Bright field images of HCE-T cells exposed to desiccation stress, with and without chloroquine (CQ) /cyclosporine (CsA) treatment under the 10X. ((A) Non-desiccated/control HCE-T cells, (B) desiccated HCE-T cells, (C) desiccated cells treated with CQ, and (D) desiccated cells treated with CsA) (Figure 1(a)). (b) Percentage of cell viability of HCE-T cells exposed to desiccation cells with and without (CQ/CsA) treatment (Figure 1(b)). (c) Bright field images of primary HCF cells exposed to desiccation stress, with and without chloroquine (CQ)/cyclosporine (CsA) treatment under the 10X objective. ((A) Non-desiccated/control HCF cells, (B) desiccated HCF cells, (C) HCF cells treated with CQ, (D) desiccated HCF cells treated with CQ, (E) HCF cells treated with CsA, and (F) desiccated cells treated with CsA) (Figure 1(c)). (d) Percentage of cell viability of HCF cells exposed to desiccation cells with and without (CQ/CsA) treatment (Figure 1(d)). (e) Bright field images of primary HCE cells exposed to desiccation stress, with and without chloroquine (CQ)/cyclosporine (CsA) treatment under the 10X objective. ((A) Non-desiccated/control primary HCE cells, (B) desiccated primary HCE cells, (C) primary HCE cells treated with CQ, (D) desiccated cells treated with CQ, (E) primary HCE cells treated with CsA, and (F) desiccated cells treated with CsA (Figure 1(e)). (f) Percentage of cell viability of primary HCE cells exposed to desiccation stress with and without (CQ/ CsA) treatment (Figure 1(f)). Data are the mean ± SD, n = 3; statistical significance was denoted (^*∗*^p < 0.05, ^*∗∗*^p < 0.01, ^*∗∗∗*^p < 0.001, and ns-nonsignificant as compared to desiccated cells). Note that --- is control, +-- are HCE/HCF cells exposed to desiccation (Des), -+- are HCE/HCF cells treated with chloroquine (CQ), ++- are desiccated HCE/HCF cells treated with chloroquine (CQ), --+ are HCE/HCF cells treated with Cyclosporine (CsA), and -++ are desiccated HCE/HCF cells treated with Cyclosporine (CsA).

HCE-T cells, primary HCF, and HCE cells under desiccation stress showed changes in morphology (shape and size) with reduction in cell viability. HCE-T cells exposed to desiccation stress showed a reduced cell viability of (80-85%) at 24 hours compared to non-desiccated cells. Desiccated cells treated with CQ showed (92-94%) and CsA (88-90%) viability with no significant morphological changes were noted at 24 hrs (Figures 1(a) and 1(b)). In addition, HCF cells with desiccation stress showed a viability of (75-80%) at 24 hours compared to non-desiccated cells and cells treated with CQ (84-86 %) and CsA showed viability (80-83%) (Figures 1(c) and 1(d)). Further, primary HCE cells under desiccation stress showed a reduction in viability of (69-73%) at 24 hours compared non-desiccated cells. Further, cells treated with CQ and CsA showed a percentage of viability of 85-90% and 83-87% in comparison with desiccated cells alone (Figures 1(e) and 1(f)).

### 3.4. CQ Reduces Desiccation Stress Induced Inflammation in HCE and HCF Cells

**Figure 2:**
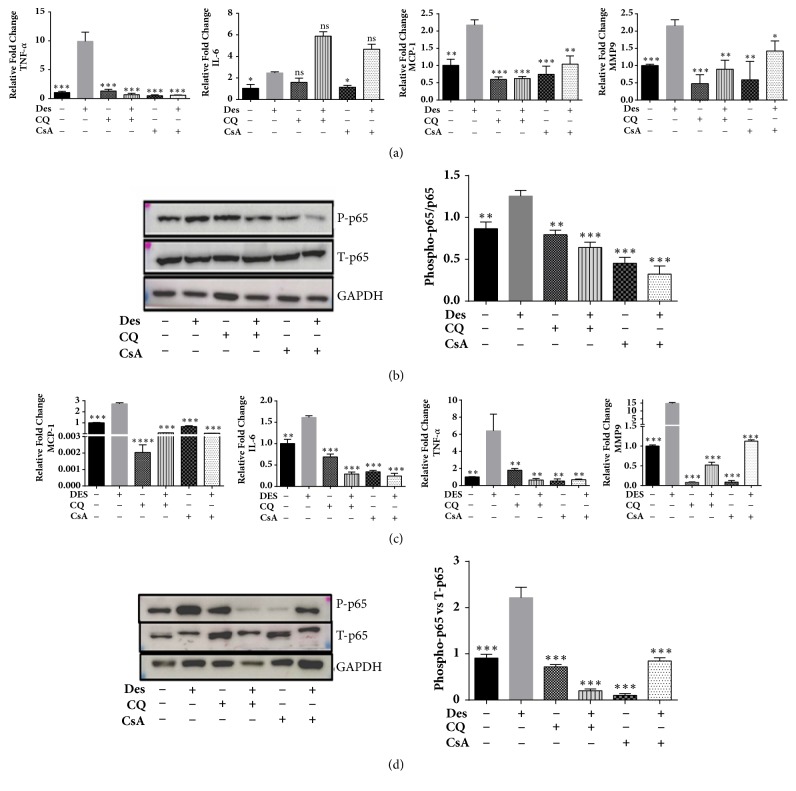
**Expression of inflammatory related genes and NF**κ**B pathway proteins in HCE-T and HCF cells under desiccation stress**. (a) The mRNA expression levels of MCP-1, MMP-9, IL-6, and TNF-*α* in HCE-T cells under desiccation stress (Figure 2(a)). (b) Immunoblot shows the phosphorylation status of p65 in HCE-T cells exposed to desiccation stress, with and without CQ/CsA treatment. Densitometric analysis of the blots showed the ratios of total p65 and phosphorylated p65 at (Ser536) (Figure 2(b)). (c) The mRNA expression levels of MCP-1, MMP-9, IL-6, and TNF-*α* in HCF cells under desiccation stress (Figure 2(c)). (d) Immunoblot shows the phosphorylation status of p65 in HCF cells exposed to desiccation with and without CQ/CsA treatment. Densitometric analysis of the blots showed the ratios of total p65 and phosphorylated p65 at (Ser536) (Figure 2(d)). Data are the mean ± SD, n = 3; statistical significance was denoted (^*∗*^p < 0.05, ^*∗∗*^p < 0.01, ^*∗∗∗*^p < 0.001, and ns- nonsignificant as compared to desiccated cells). Note that --- is control, +-- are HCE-T/HCF cells exposed to desiccation (Des), -+- are HCE-T/HCF cells treated with chloroquine (CQ), ++- are desiccated HCE-T/HCF cells treated with chloroquine (CQ), --+ are HCE-T/HCF cells treated with Cyclosporine (CsA) and -++ are desiccated HCE-T/HCF cells treated with cyclosporine (CsA).

HCE-T and HCF cells under desiccation stress with and without CQ, CsA treatment were analyzed for the expression of inflammation related genes IL6, TNF-*α*, MCP-1, and MMP-9 by using qPCR. The mRNA levels of IL6, TNF-*α*, MCP-1, and MMP-9 were increased in HCE-T and HCF cells exposed to desiccation stress compared to control (Figures 2(a) and 2(c)). In addition, cells treated with CQ and CsA showed decrease in levels of TNF-*α*, MCP-1, and MMP-9 compared to desiccated cells. Western blotting was carried out to analyse the activation status of p65 (phosphorylation at ser536). An increased phosphorylation level of p65 was observed in desiccated cells compared to control (Figures 2(b) and 2(d)). Further, cells treated with CQ and CsA showed decrease in phosphorylation levels of p65 in comparison to desiccated cells (Figures 2(b) and 2(d)).

### 3.5. Regulation of NF*κ*B Pathway by CQ in HCE-T Cells under Desiccation Stress

**Figure 3:**
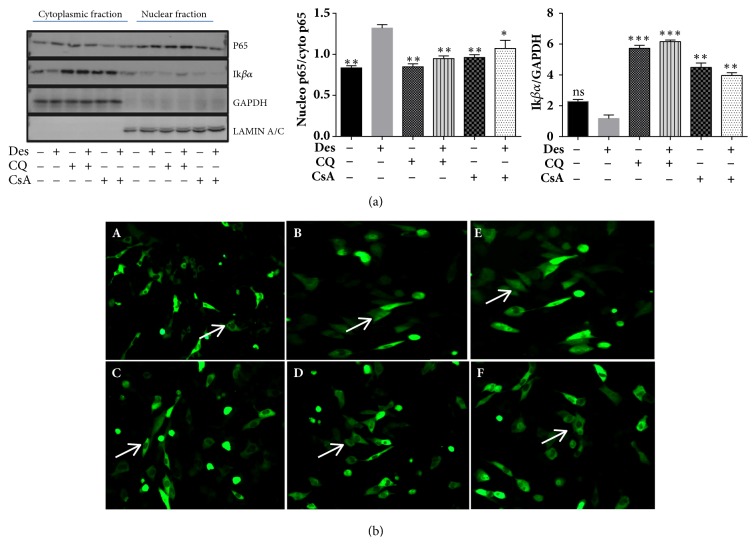
**Expression of p65 and I**κ**B**α** proteins in HCE-T cells under desiccation stress**. (a) Immunoblots of nuclear/cytoplasmic fractions shows protein expression levels of p65 (NF*κ*B) and I*κ*B*α* in HCE-T cells exposed to desiccation stress (treated with or without CQ and CsA). Densitometric analysis of the blots shows the ratios of total p65 to I*κ*B*α* (Figure 3(a)). (b) GFP-RelA translocation images at 20X magnification of HCE-T cells under desiccation stress treated with/without CQ and CsA (Figure 3(b)). ((A) Non-desiccated/control HCE-T cells, (B) desiccated HCE-T cells, (C) desiccated cells treated with CQ, (D) desiccated HCE-T cells treated with CsA, (E) HCE-T cells treated with TNF-*α* (10 ng/ml), and (F) HCE-T cells treated with TNF-*α* (10 ng/ml)+CsA. Data are the mean ± SD values, n = 3, statistical significance was denoted (^*∗*^p < 0.05, ^*∗∗*^p < 0.01, ^*∗∗∗*^p < 0.001, ns- nonsignificant as compared to levels of desiccated cells). Note that --- is control, +-- HCE-T cells exposed to desiccation (Des), -+- HCE-T cells treated with chloroquine (CQ), ++- desiccated HCE-T cells treated with chloroquine (CQ), --+ HCE-T cells treated with cyclosporine (CsA), and -++ desiccated HCE-T cells treated with cyclosporine (CsA).

The NF*κ*B pathway regulation by CQ was analysed in nuclear/cytoplasmic fractions of HCE-T cells subjected to desiccation stress. The results revealed an increased p65 level in nuclear compared to the cytoplasmic fraction in HCE-T cells exposed to desiccation stress (Figure 3(a)). In contrast, HCE-T cells treated with CQ and CsA showed increased retention of p65 in cytoplasm compared to desiccated cells without treatment. Further, the cytoplasmic I*κ*B*α* levels in HCE-T cells exposed to desiccation stress with and without CQ/CsA treatment were analysed by western blotting (Figure 3(a)). Desiccated cells showed decreased I*κ*B*α* levels compared to control. Whereas, desiccated cells treated with CQ exhibited higher levels of I*κ*B*α* compared to desiccated cells (Figure 3(a)). These results were further confirmed by monitoring GFP-RelA (p65) nuclear translocation in HCE-T cells exposed to desiccation stress with and without CQ and CsA treatment. Desiccated/TNF-*α* treated cells showed increased GFP-RelA nuclear translocation compared to control (Figure 3(b)). Besides, treatment with CQ/CsA showed reduced or partial levels of GFP-RelA nuclear translocation compared to desiccated cells (Figure 3(b)).

### 3.6. Induction of Autophagy in HCE-T Cells Exposed to Desiccation Stress

**Figure 4:**
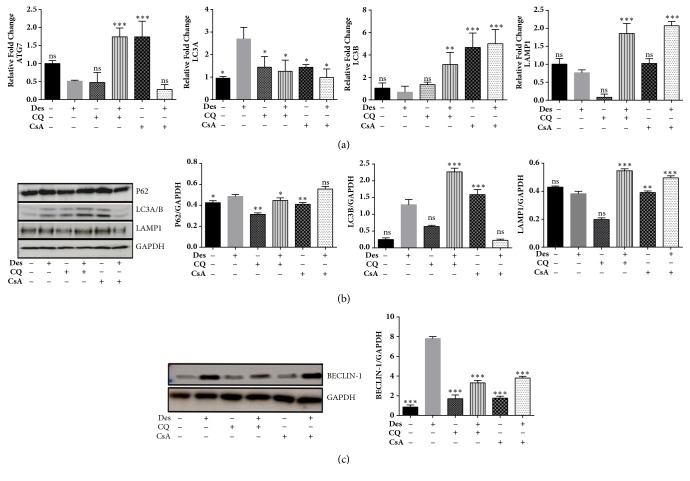
**(a)-(c) Expression of autophagy related genes and proteins in HCE-T under desiccation stress**. (a) The mRNA expression levels of LC3A, LC3B, ATG7, and LAMP1 in HCE-T cells exposed to desiccation stress, treated with and without CQ/CsA treatment normalized with *β*-actin (Figure 4(a)). (b) Immunoblot shows the protein levels of LC3, p62, and LAMP1 HCE-T cells exposed to desiccation stress and treated with and without CQ/CsA treatment (Figure 4(b)). Densitometric analysis of the blots showed the ratios of LAMP1, LC3-II and p62 to GAPDH. (c) Westernblot shows the expression levels of Beclin-1 protein in HCE-T cells exposed to desiccation stress and treated with and without CQ/CsA treatment (Figure 4(c)). Densitometric analysis of the blots showed the ratios of Beclin-1 to GAPDH. Data are the mean ± SD values, n = 3, statistical significance denoted (^*∗*^p < 0.05, ^*∗∗*^p < 0.01, ^*∗∗∗*^p < 0.001, and ns- nonsignificant as compared to levels of desiccated cells). Note that --- is control, +-- HCE-T cells exposed to desiccation (Des), -+- HCE-T cells treated with chloroquine (CQ), ++- desiccated HCE-T cells treated with Chloroquine (CQ), --+ HCE-T cells treated with cyclosporine (CsA), and -++ desiccated HCE-T cells treated with cyclosporine (CsA).

**Figure 5:**
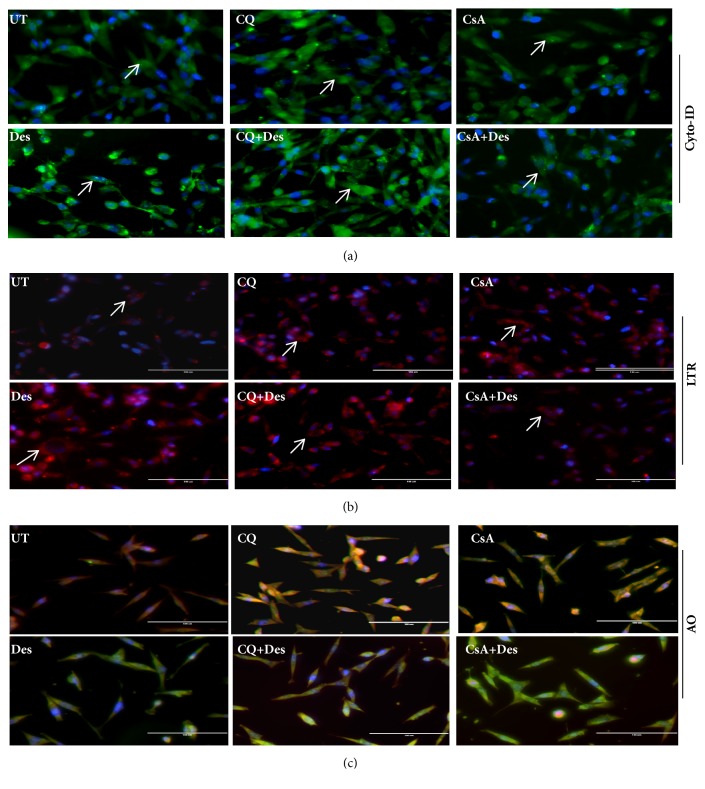
**(a)-(c) Quantification of autophagosomes and lysosomes in desiccated HCE-T cells**. (a) Cyto-Id staining for quantification of autophagosome staining in HCE-T cells under desiccation stress, with and without CQ/CsA treatment (Figure 5(a)). (b) LTR- lysotracker red staining to measure lysosome levels staining in HCE-T cells under desiccation stress, with and without CQ/CsA treatment (Figure 5(b)). (c) AO- Lysosomal pH assessed using acridine orange (AO) staining in HCE-T cells under desiccation stress, with and without CQ/CsA treatment (Figure 5(c)). UT- control or undesiccated cells, Des- HCE-T cells exposed to desiccation, CQ- HCE-T cells treated with chloroquine (CQ), CQ+Des- desiccated HCE-T cells treated with chloroquine (CQ), CsA- HCE-T cells treated with cyclosporine (CsA), and CsA+Des- desiccated HCE-T cells treated with cyclosporine (CsA).

HCE-T cells exposed to desiccation stress with and without CQ/CsA treatment was analysed for autophagy related markers by measuring the levels of mRNA, protein, autophagosomes and lysosomes. The mRNA expression level of autophagosomal marker LC3A was significantly higher in desiccated cells compared to control, whereas there were no changes in the levels of LC3B, ATG7, and LAMP1(lysosomal marker) (Figure 4(a)). Further, cells treated with CQ/CsA showed increase in levels of LC3B, ATG7 and LAMP1 compared to desiccated cells (Figure 4(a)). Western blotting showed an increase in the expression levels of LC3B and p62 with no changes in the LAMP1 levels in HCE-T cells exposed to desiccation stress compared to control (Figure 4(b)). Cells treated with CQ showed increase in LC3B and LAMP1 levels compared to desiccated cells, whereas HCE-T treated with CsA showed increased levels of LAMP1 and p62 with decreased levels of LC3B compared to desiccated cells (Figure 4(b)). These results were further confirmed by Cyto-ID and Lysotracker red (LTR) which were used to monitor the levels of autophagosomes and lysosomes in HCE-T cells exposed to desiccation stress with andwithout CQ/CsA treatment. Increased levels of autophagosomes (green fluorescence) and lysosomes (red fluorescence) were observed in HCE-T cells exposed to desiccation stress compared to control in the perinuclear regions (Figures 5(a) and 5(b)). Additionally, desiccated cells treated with CQ showed an increased number of autophagosome and lysosome, but treatment with CsA did not show any significant changes in autophagosome/lysosome levels. Acridine orange dye was used to analyse intracellular lysosome pH in HCE-T cells exposed to desiccation stress. It was observed that desiccated cells showed change in lysosomal pH compared to control (red to green shift). CQ and CsA were able to maintain the pH in comparison to desiccated cells (Figure 5(c)).

### 3.7. Analysis of the Effect of CQ on Signalling Pathways Associated with Desiccation Stress

**Figure 6:**
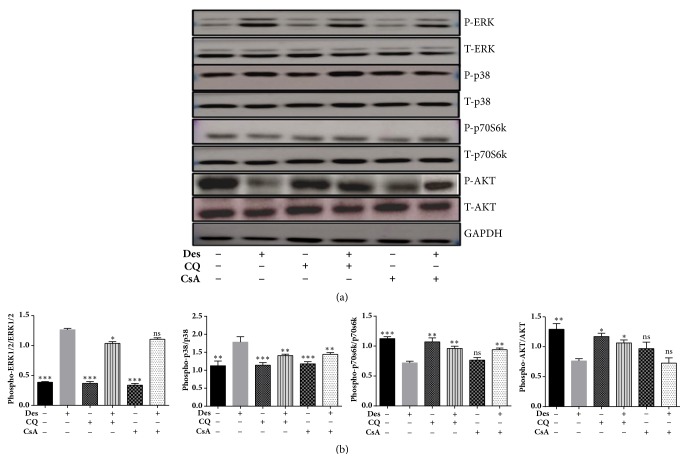
**Phosphorylation levels of MAP kinase, AKT/p70S6kinase and AMPK proteins in HCE-T cells under desiccation stress**. Immunoblot shows phosphorylated ERK1/2, p38, AKT and P70S6kinase, and AMPK (Figure 6). Densitometric analysis of the blots showed the ratios of phosphorylated AKT, p70s6kinase, ERK1/2 and p38 to AKT, p70s6kinase, ERK1/2, and p38. Data are the mean ± SD values, n = 3, statistical significance denoted (^*∗*^p < 0.05, ^*∗∗*^p < 0.01, ^*∗∗∗*^p < 0.001, and ns- nonsignificant as compared to desiccated cells). Note that --- is control, +-- HCE-T cells exposed to desiccation (Des), -+- HCE-T cells treated with chloroquine (CQ), ++- desiccated HCE-T cells treated with chloroquine (CQ), --+ HCE-T cells treated with cyclosporine (CsA), and -++ desiccated HCE-T cells treated with cyclosporine (CsA).

To elucidate the pathways regulated by CQ in HCE-T cells under desiccation stress, immunoblotting was performed for evaluating the phosphorylation levels of MAPKinase, AKT/p70S6kinases and AMPK proteins. HCE-T cells under desiccation stress showed increased phosphorylation levels of ERK1/2 at Thr180/Thy204 and p38 (Thr180/Thy182) proteins (Figure 6), along with a decrease in the phosphorylation levels of AKT (Ser473) and p70S6kinase (Thy389) compared to control. Interestingly, treatment with CQ/CsA decreased the phosphorylation levels of ERK1/2 (Thr180/Thy204) and p38 (Thr180/Thy182), along with an increase in phosphorylation of levels AKT (Ser473)/p70S6kinase (Thy389), compared to desiccated cells (Figure 6).

## 4. Discussion

In the present study, HCE and HCF cells exposed to desiccation stress were used as an experimental model for studying the pathogenesis dry eye disease. The therapeutic effect of CQ in human corneal cell types exposed to desiccation stress was evaluated by examining the inflammation and autophagy related molecular factors. We found that HCE-T cells, primary HCF and HCE cells under desiccation stress exhibited 15-30% decrease in cell viability compared to control. Desiccation stress has been used as an experimental condition for studying the pathogenesis of dry eye and studies have reported that desiccation causes intracellular changes leading to apoptosis [21, 22]. Cytomorphological changes were noted in the corneal epithelium comprising changes in size of cells and the occurrence of goblet cells with tubular, tortuous chromatin during desiccation stress [23–25]. Similarly, we observed that HCE-T, primary HCF and HCE cells exposed to desiccation stress showed morphological changes in the cell size and shape compared to control. Dry eye disease is characterized by ocular inflammation; clinical studies have reported elevated levels of IL-1, IL-6, TNF-*α*, and TGF-*β*1 [26–28], along with MMP-9 in the tear film of dry eye patients [29, 30].* In vitro* and* in vivo* studies on HCE cells and mouse corneal epithelium cells have shown increased expression of IL-6, IL-8, TNF-*α*, MMP's, and IL-8 levels on exposure to desiccation stress [31, 32]. It was seen that desiccation stress in HCE-T cells led to an increase in the expression levels of IL-6, TNF-*α*, MCP-1, and MMP-9 genes compared to control, which is in agreement with previous findings [32]. In the present study, we found that desiccation stress induces inflammation in HCE-T and HCF cells which was regulated through canonical NF-*κ*B pathway. Activation and nuclear translocation of NF-*κ*B/RelA leads to the transcription of downstream pro-inflammatory genes [33]. There was a significant increase in the phosphorylation level of p65 followed by its nuclear translocation in desiccated cells, which could have led to activation of the downstream targets IL6, TNF-*α*, MMP-9, and MCP-1. Autophagy has been associated with inflammation [34] and studies have reported the induction of autophagy during desiccation stress as an adaptive response which leads to cell survival or cell death in yeast [35]. Increased expression of two major autophagy markers, LC3 (autophagosomes) and LAMP1 (lysosomes), was seen in HCE-T cells exposed to desiccation stress, which may indicate the induction of autophagy during desiccation, as an adaptive response.Further, it is well known that dephosphorylation of AKT/p70S6K is associated with mTOR mediated induction of autophagy [36]. Interestingly, we observed dephosphorylation in the levels of AKT/p70S6k in HCE-T cells exposed to desiccation stress, which may also suggest the induction of autophagy during desiccation. Additionally, induction of autophagy may also lead to autophagy mediated cell death, which requires regulators such as beclin1. Studies have reported that knockdown of beclin1 gene protects neuronal cells against ischemia indicating autophagy mediated cell death [37]. We found elevated levels of beclin 1 in desiccated HCE-T cells, which may indicate the possibility of autophagy mediated cell death as a consequence of desiccation stress. The increased number of autophagosomes (LC3) and lysosomes (LAMP1) in desiccated HCE-T cells treated with CQ suggest that there was no change in desiccation mediated autophagic flux [20]. On the other hand, this would be possible because the formulated concentration of CQ might not affect the autophagic flux rate in HCE-T cells under desiccation stress.

Choloroquine is used as an anti-inflammatory drug for treating chronic disease such as rheumatoid arthritis [5]. Experimental studies have illustrated that chloroquine blocks LPS induced expression of TNF-*α* and IL-6 in macrophages and PBMC's [38–40]. It was also found to reduce expression of IL-1 and cell-associated TNF-*α* in LPS induced inflammation [41, 42]. Similarly, our results revealed that treatment with CQ decreased the expression levels of inflammatory genes TNF-*α*, MCP-1 and MMP-9 in HCE-T and HCF cells exposed to desiccation stress. Our data additionally demonstrate that CQ regulates NF*κ*B by inhibiting the phosphorylation and nuclear translocation of p65/RelA, thereby blocking the transcription of downstream targets.

Our data further reveals an increased phosphorylation levels of ERK1/2 and p38 proteins in HCE-T cells exposed to desiccation stress, which might be an adaptive response of HCE-T cells to desiccation stress. Studies have reported that activation of MAPK proteins- ERK1/2 and p38 during desiccation stress [43, 44]. Increased phosphorylation of p38 in desiccated HCE-T cells might be involved in the reduced cell viability since it has been reported that prolonged phosphorylation of p38 leads to cell death in HEK-293 cells exposed to hyperosmolarity stress [45]. Interestingly, treatment with CQ reduced the phosphorylation levels of p38 thereby reducing cell death in HCE-T cells exposed to desiccation stress.

Induction of pro-survival pathways during desiccation stress helps mammalian cells to attain tolerance towards stress [46]. We observed that treatment of CQ was able increase the phosphorylation levels of AKT/p70S6k proteins thereby preventing apoptosis in HCE-T cells exposed to desiccation stress. Further, it has been shown that upstream (AKT) and downstream targets (p70S6k) of mTOR play a key role in regulating apoptosis and autophagy in UV induced cell death [47]. CQ acts as an anti-inflammatory agent through its lysomotrophic [48] or nonlysomotrophic activity [42]; henceforth in our future studies we would like to explore on the anti-inflammatory activity of the CQ is attributed to its lysomotrophic and nonlysomotrophic action. While it is a limitation of the present study, it would be interesting to analyse the effect of CQ preclinical and clinical studies, which may further validate the anti-inflammatory and cytoprotective effect of CQ.

It has been shown that CQ at high prolonged doses causes severe systemic and nonsystemic side effects, whereas the low does and application have not shown any side effects [11]. These side effects might be consequences of autophagic blocking activity of CQ at high doses. Therefore, we hypothesise that low concentrations CQ acts as an anti-inflammatory agent without changes in autophagy dynamics in human corneal epithelial and fibroblast cells.

## 5. Conclusion

In conclusion, CQ protects human corneal cells from desiccation stress induced inflammation through its interaction with the canonical NF*κ*B pathway. Activation of autophagy and cell death in HCE-T cells under desiccation stress might be mediated through the mTOR/MAPKinase and pathways. CQ regulated the phosphorylation MAPKinase and mTOR related proteins, thereby preventing desiccation stress induced cell death, without altering the autophagy flux. These results suggest that CQ may be used as an alternate therapy for inflammation associated with dry eye disease.

## Figures and Tables

**Table 1:** Primers used for quantitative-qPCR analysis.

**Gene Name**	**Sequences (5'-3')**	**Gene acc no**
**LC3A**	FP: CGTCCTGGACAAGACCAAGT RP: CTCGTCTTTCTCCTGCTCGT	NM_032514

**LC3B**	FP:AGCAGCATCCAACCAAAATC RP:CTGTGTCCGTTCACCAACAG	NM_022818

**LAMP1**	FP: AGTGGCCCTAAGAACATGACC RP: AGTGTATGTCCTCTTCCAAAAGC	NM_005561

**MMP9 **	FP: GGGCTTAGATCATTCCTCAGTG RP: GCCATTCACGTCGTCCTTAT	NM_004994

**LOX**	FP: ACATTCGCTACACAGGACATC RP: TTCCCACTTCAGAACACCAG	NM_002317

**IL6**	FP: GATGAGTACAAAAGTCCTGATCCA RP: CTGCAGCCACTGGTTCTGT	NM_54894

**ATG7**	FP: GGA TGA AGC TCC CAA GGA CAT RP: CCA GCA GAG TCA CCA TTG TAG TA	NM_001144912

**TNF-**α	FP: CAGCCTCTTCTCCTTCCTGAT RP: GCCAGAGGGCTGATTAGAGA	NM_000594

β** ACTIN**	FP: GCCAACCGCGAGAAGATGA RP: CCATCACGATGCCAGTGGTA	NM_001101

## Data Availability

The data used to support the findings of this study are available from the corresponding author upon request.

## Abbreviations

DED: Dry eye disease
CQ: Chloroquine
CsA: Cyclosporine.

## Supplementary Materials

Supplementary Materials Supplementary Figure 1: Cytotoxicity effect of CQ in HCE-T cells. Supplementary Figure 2: Immunostaining of Cytokeratin 3 in HCE cells.
