# Supplementary material for: Chloroquine Protects Human Corneal Epithelial Cells from Desiccation Stress Induced Inflammation without Altering the Autophagy Flux
Source: Biomed Res Int. 2018 Nov 1;2018:7627329. doi: 10.1155/2018/7627329 (PMC6241345; doi:10.1155/2018/7627329)
Supplement: Supplementary Materials — Supplementary Figure 1: Cytotoxicity effect of CQ in HCE-T cells. Supplementary Figure 2: Immunostaining of Cytokeratin 3 in HCE cells. [file 7627329.f1.zip › 7627329.f1/supplementary data file _18-10-18_BMRI_2524177.docx]

**Supplementary data**

**1. Materials and methods**

***1.1 Viability assay for HCE-T cells treated with CQ***

HCE-T cells were treated with different concentrations (0.00006 to 0.003%) of CQ drops for 48hrs. Tryphan blue assay was used to determine the cell viability.

**2 Results**

***2.1 Cytotoxic activity of CQ drops on HCE-T cells***

HCE-T cells were treated with different concentrations of CQ drops (0.00006 to 0.003%) for 48 hrs. The Cells treated with 0.00006% of CQ showed 3 % cell death and 80-84 % was observed at 0.003% (Supplementary figure 1).

**Supplementary Figure 1**


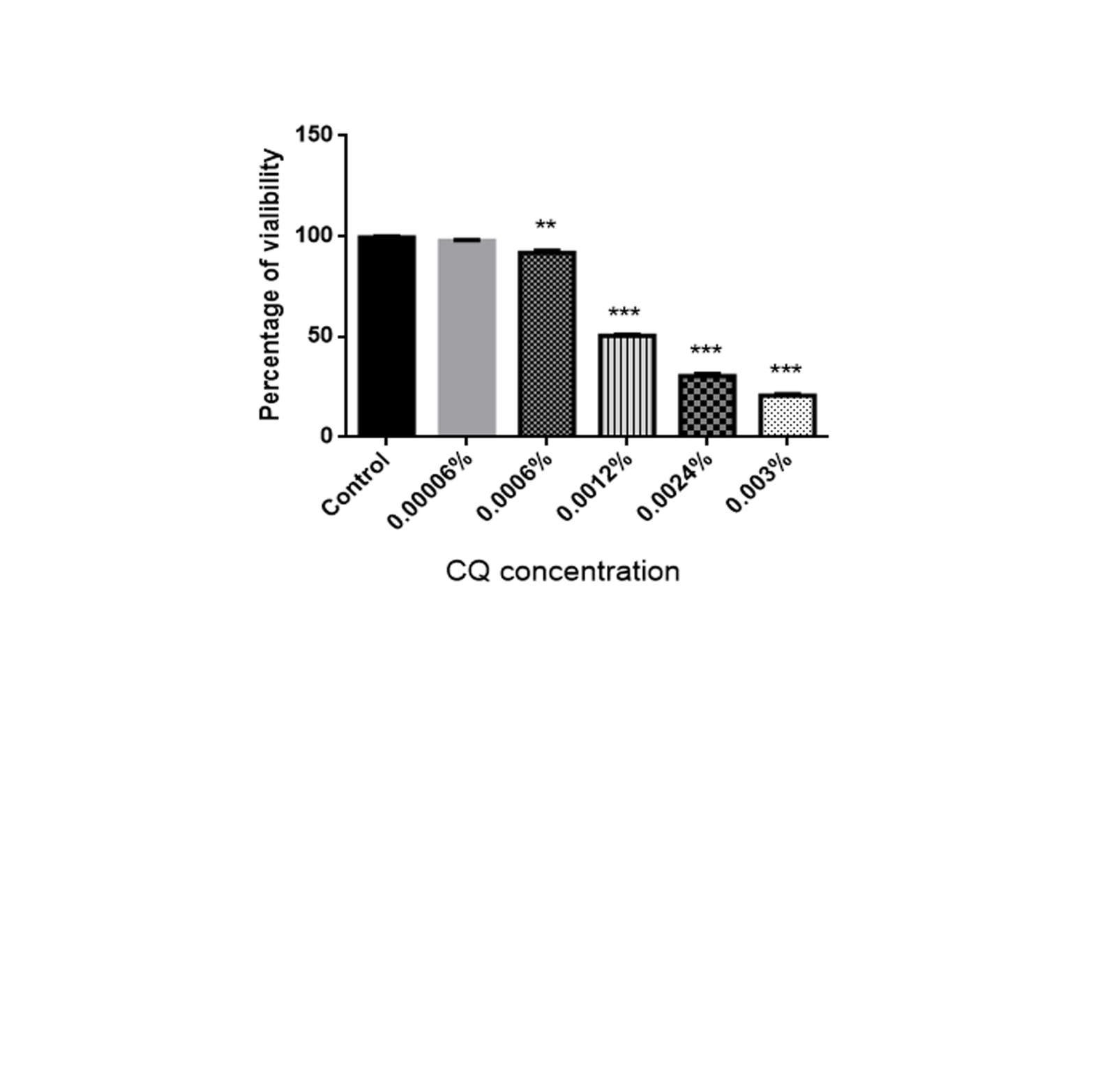


**Cytotoxicity effect of CQ in HCE-T cells**

Bar graph representing the percentage of viable cells treated with different concentrations of CQ drops for 48 hrs. Data are the mean ± SD values, n=3, statistical significance denoted as (**p < 0.01, *p < 0.05 compared to control cells).

**Immunofluorescence staining**

HCE-T cells were cultured on chamber slides at density of 0.1X10^6^ cells/well. After 24 hours the media was removed and cells were fixed with 100% ice cold methanol for 5 minutes at room temperature. Further cells were treated with permeabilization buffer containing 1XPBS and 0.1% triton X-100. Cells were then blocked with 3% bovine serum albumin (BSA) at room temperature for 30 minutes, followed by incubation with primary cytokeratin 3 antibody (1:500) overnight at 4 degree. Alexa fluor 488- conjugated anti mouse secondary antibody (abcam, Cat no- ab150113) was used (1:2000) and kept for 1 hour incubation at room temperature. Finally the cells were mounted using fluoroshield containing DAPI (Fluorosheild^TM^ sigma, cat no-76057). Cells were examined under fluorescent microscope using FL1 and FL2 channels.

***2. Results***

***2.2 Cytokeratin 3 staining in HCE cells***

Immunostaining of cytokeratin 3 in HCE cells showed presence of green fluorescence in the cytoplasm region, with the typical sign of filamentous staining emerging from the nucleus to cell membrane.

***Supplementary Figure 2***

**Immunostaining of Cytokeratin 3 in HCE cells**


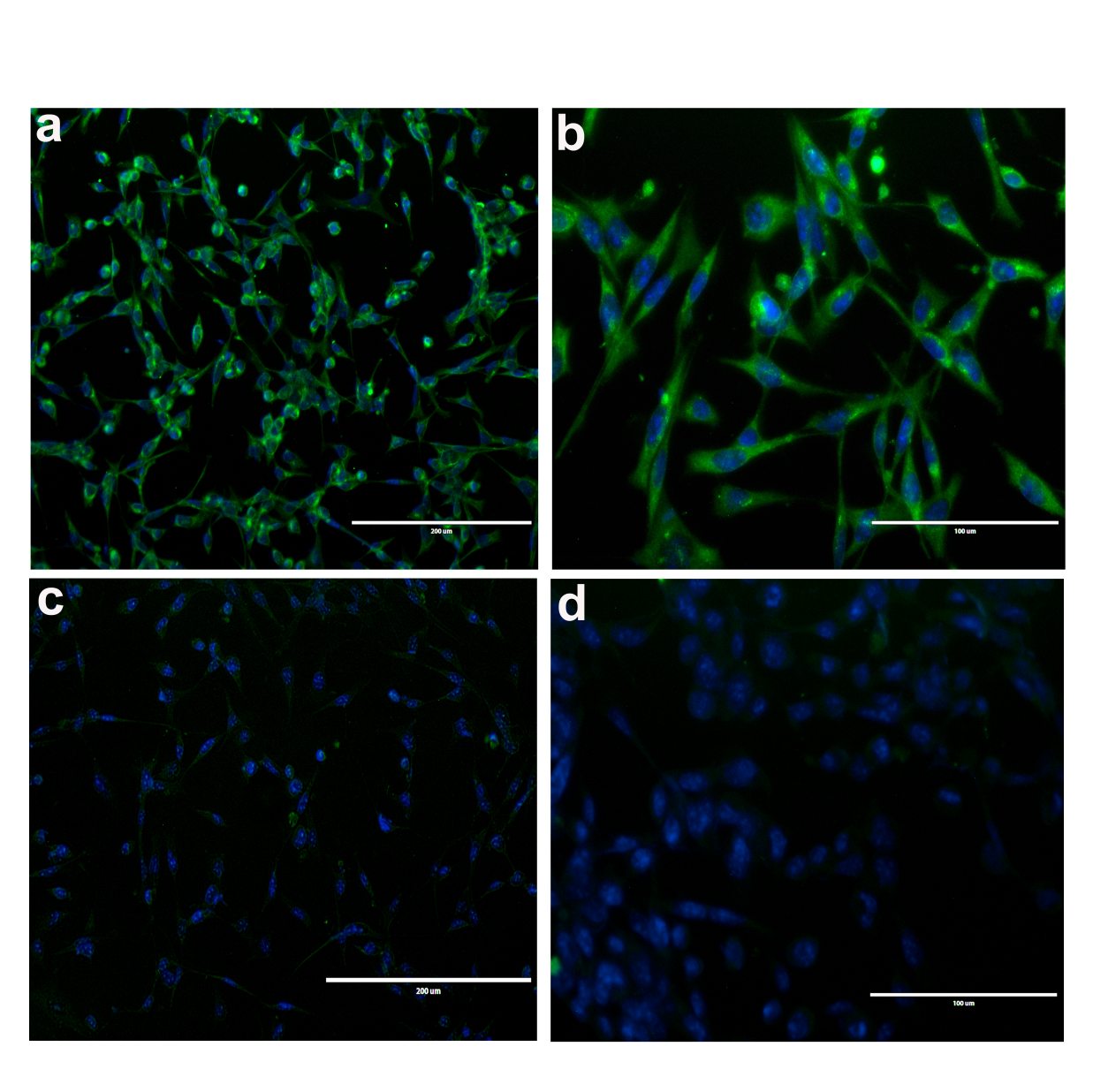


(a, b)- HCE cells stained with CK3 showed presence of green fluorescence in cytoplasmic region and nucleus stained with DAPI (Blue).

(c, d)- HCE cells stained Alexa Fluor 488 (secondary antibody alone) and DAPI (nuclear stain- blue) as a negative control.

Images were acquired at 20X, 40X magnification.
