# Supplementary material for: Chloroquine Protects Human Corneal Epithelial Cells from Desiccation Stress Induced Inflammation without Altering the Autophagy Flux
Source: Biomed Res Int. 2018 Nov 1;2018:7627329. doi: 10.1155/2018/7627329 (PMC6241345; doi:10.1155/2018/7627329)
Supplement: Supplementary Materials — Supplementary Figure 1: Cytotoxicity effect of CQ in HCE-T cells. Supplementary Figure 2: Immunostaining of Cytokeratin 3 in HCE cells. [file 7627329.f1.zip › 7627329.f1/supplementary data file _18-10-18_BMRI_2524179.pdf]

Supplementary data

1. Materials and methods

1.1 Viability assay for HCE-T cells treated with CQ

HCE-T cells were treated with different concentrations (0.00006 to 0.003%) of CQ drops for 48hrs. Tryphan blue assay was used to determine the cell viability.

2 Results

2.1 Cytotoxic activity of CQ drops on HCE-T cells

HCE-T cells were treated with different concentrations of CQ drops (0.00006 to 0.003%) for 48 hrs. The Cells treated with 0.00006% of CQ showed 3 % cell death and 80-84 % was observed at 0.003% (Supplementary figure 1).

Supplementary Figure 1

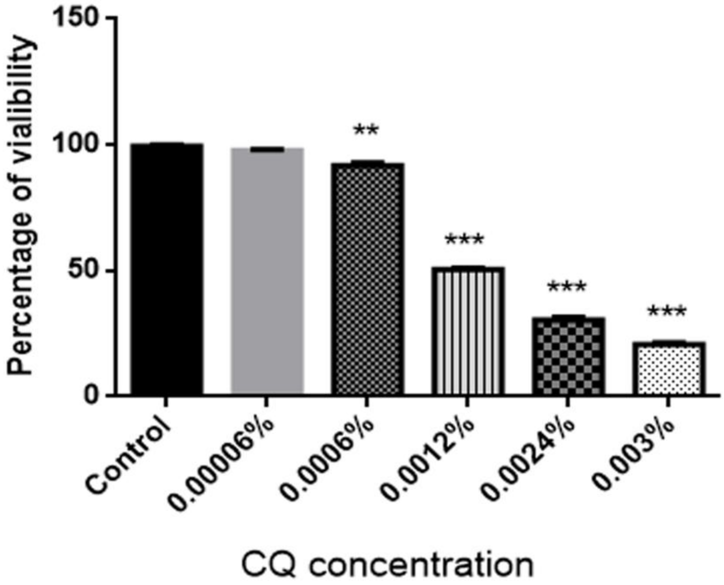

Cytotoxicity effect of CQ in HCE-T cells

Bar graph representing the percentage of viable cells treated with different concentrations of CQ drops for 48 hrs. Data are the mean ± SD values, n=3, statistical significance denoted as (**p < 0.01, *p < 0.05 compared to control cells).

Immunofluorescence staining

HCE-T cells were cultured on chamber slides at density of $0.1X10^6$ cells/well. After 24 hours the media was removed and cells were fixed with 100% ice cold methanol for 5 minutes at room temperature. Further cells were treated with permeabilization buffer containing 1XPBS and 0.1% triton X-100. Cells were then blocked with 3% bovine serum albumin (BSA) at room temperature for 30 minutes, followed by incubation with primary cytokeratin 3 antibody (1:500) overnight at 4 degree. Alexa fluor 488- conjugated anti mouse secondary antibody (abcam, Cat no- ab150113) was used (1:2000) and kept for 1 hour incubation at room temperature. Finally the cells were mounted using fluoroshield containing DAPI (Fluorosheild™ sigma, cat no-76057). Cells were examined under fluorescent microscope using FL1 and FL2 channels.

2. Results

2.2 Cytokeratin 3 staining in HCE cells

Immunostaining of cytokeratin 3 in HCE cells showed presence of green fluorescence in the cytoplasm region, with the typical sign of filamentous staining emerging from the nucleus to cell membrane.

Supplementary Figure 2

Immunostaining of Cytokeratin 3 in HCE cells

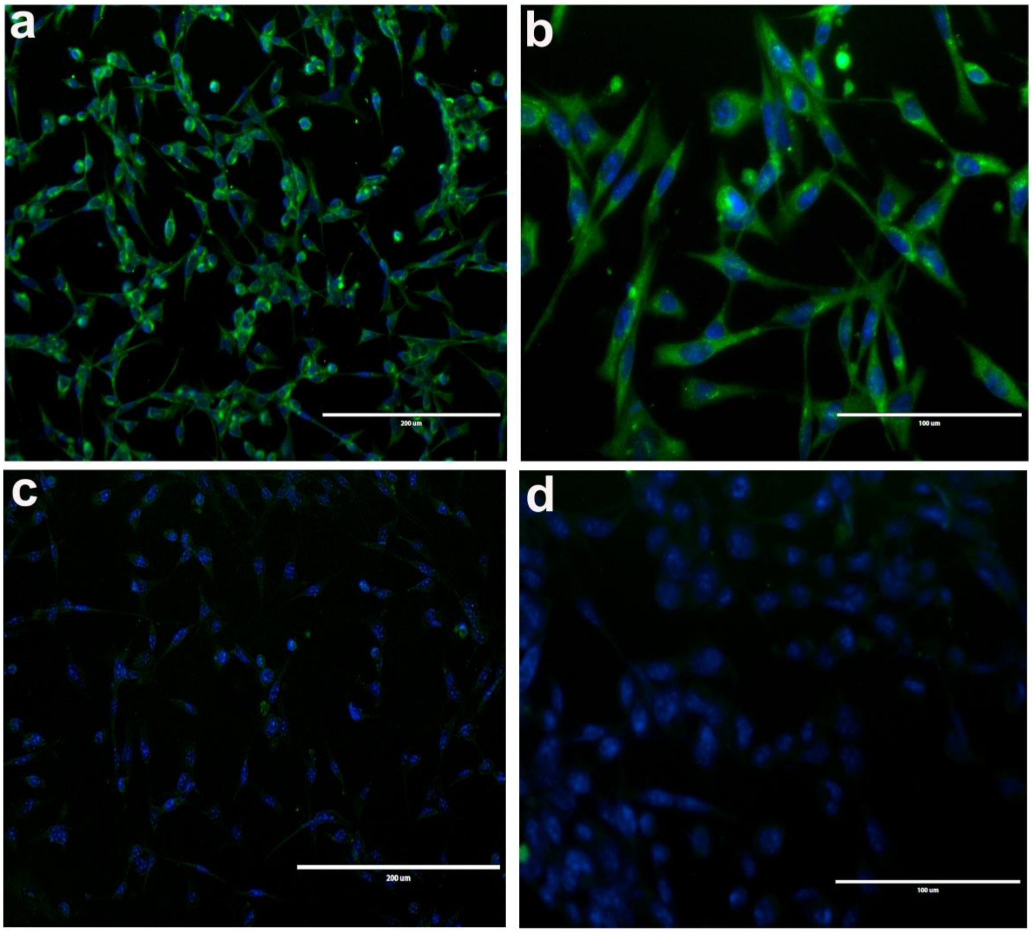

(a, b)- HCE cells stained with CK3 showed presence of green fluorescence in cytoplasmic region and nucleus stained with DAPI (Blue).

(c, d)- HCE cells stained Alexa Fluor 488 (secondary antibody alone) and DAPI (nuclear stain- blue) as a negative control.

Images were acquired at 20X, 40X magnification.
